# Screening of genes associated with early stages of adventitious root formation from progenitor adult cells of pine

**DOI:** 10.1186/1753-6561-5-S7-P133

**Published:** 2011-09-13

**Authors:** Dolores Abarca, Alberto Pizarro, Alicia Del Amo, Carmen Diaz-Sala

**Affiliations:** 1 Department of Plant Biology, University of Alcala, 28871 Alcalá de Henares, Madrid. Spain

## Background

In plants, the possibility to regenerate adventitious roots, shoots or embryos directly from adult tissues has been known for more than 60 years and has been exploited in horticulture, agriculture and forestry [1]. However, little is known about the mechanisms that enable a somatic differentiated cell to switch its fate into a multipotent, pluripotent or totipotent cell that can develop a root, shoot or embryo or repair damaged tissues [2]. Although apparent dedifferentiation and respecification of cells seems to occur, whether acquisition of competence to regenerate organs occurs, as in animal cells, through dedifferentiation, or whether it is via transdifferentiation or by the use of pre-existent totipotent, pluripotent or multipotent cells in adult tissues remains unknown. In either case, cell fate switches are characterized by remarkable changes in the pattern of gene expression, as cells switch from an expression pattern typical of a somatic cell to a new one directing a new developmental pathway [3]. Thus, determining the way by which cells reset their gene expression pattern,especially for the timetable and repertoire of gene expression characteristic of the earliest stages of normal development, is crucial to understand cellular plasticity.

In forest species, a loss of regeneration capacity is associated with tree age and maturation, which makes forest species representative and reliable systems to study how cell fate becomes fixed during development and how plant cells can manage to retain developmental plasticity [4]. The decline in the capacity to regenerate roots from cuttings is one of the most dramatic effects of tree maturation and has been the subject of investigations on the basic nature of the process [5].

## Methods

An experimental design based on the analysis of temporal and spatial expression of genes during the earliest steps of adventitious root formation, when cell dedifferentiation and reorganization takes place but before the onset of cell division, was set up in pine cuttings to provide additional clues to the process [6,7]. Two approaches were followed: 1. Expression screening using cDNA collections, and 2. Analysis of the expression pattern of candidate genes potentially involved in root meristem specification, such as genes encoding GRAS proteins, transcription factors involved not only in root patterning but also in the establishment of the quiescent centre identity and in the maintenance of the stem cell status of the surrounding initial cells during the embryonic pattern formation and postembryonary development.

## Results and conclusions

For the first approach, the expression pattern of regulatory genes identified from cDNA collections enriched in auxin-responsive, meristem specific and regeneration capacity specific genes was analysed by cDNA microarray and by qRT-PCR. Gene clustering by functional classification and expression pattern revealed the existence of auxin-dependent and auxin-independent pathways potentially involved in the initial phases of regeneration. In addition, genes specifically expressed either in competent or in non-competent tissues were identified.

For the second approach, fifteen *GRAS* genes have been identified in the conifer genome using *in silico* analysis of conifer gene databases. Expression analysis in rooting competent and non-competent pine cuttings revealed different patterns of expression during adventitious root induction: 1. genes that were induced in the presence of exogenous auxin within the initial 24 of the root induction process in rooting competent cuttings only (*PrSCL1*), 2. genes that were induced within the initial 24 of the root induction process in rooting competent cuttings only, and whose expression was not dependent on the presence of exogenous auxin (*PrSHR*), 3. genes that were not induced during the adventitious rooting but their level of expression wasassociated with the developmental stage of the cutting (*PrSCR*), 4. genes that showed a transient short-term expression in both types of cuttings, and 5. genes that did not show an expression pattern related to neither developmental stage nor adventitious rooting competence. Spatial analysis of the expression of two of these genes, *PrSCL1* and *PrSHR*, during adventitious root formation showed a transient increase of mRNA levels asymmetrically localized to the cambial area and rooting competent cells during the earliest stages of adventitious root induction, before the resumption of cell division leading to the root formation pathway, but not in non-competent cuttings. These results suggest the presence of specific cellular signalling pathways or specific factors, perhaps distributed in a localized- or developmental-specific manner, in the tissues involved in rooting. Since auxin transport, accumulation and metabolism do not account for the difference in the rooting capacity of pine cuttings, other factors such as an asymmetric auxin distribution or other determination factors could be involved in the control of age dependent cellular plasticity. Auxin distribution has been analyzed in rooting competent and non-competent cuttings during the initial 24 h of the root induction process. Results showed an asymmetric auxin distribution localized to the cambial area and rooting competent cells in rooting competent cuttings. The spatiotemporal colocalization of auxin accumulation and *GRAS* gene expression was observed in rooting competent cuttings only. This result suggests that signaling pathways involving *GRAS* transcription factors and auxin distribution could be related to the regulation of age-related changes in regeneration capacity.
